# Lessons From the Implementation of Mo-Buzz, a Mobile Pandemic Surveillance System for Dengue

**DOI:** 10.2196/publichealth.7376

**Published:** 2017-10-02

**Authors:** May Oo Lwin, Karthikayen Jayasundar, Anita Sheldenkar, Ruwan Wijayamuni, Prasad Wimalaratne, Kacey C Ernst, Schubert Foo

**Affiliations:** ^1^ Wee Kim Wee School of Communication and Information Nanyang Technological University Singapore Singapore; ^2^ Colombo Municipal Council Colombo Sri Lanka; ^3^ School of Computing University of Colombo Colombo Sri Lanka; ^4^ Epidemiology and Biostatistics Department Mel and Enid Zuckerman College of Public Health Arizona, AZ United States

**Keywords:** pandemics, dengue, health communication, telemedicine, epidemiology, participatory surveillance, participatory epidemiology

## Abstract

**Background:**

Approximately 128 countries and 3.9 billion people are at risk of dengue infection. Incidence of dengue has increased over the past decades, becoming a growing public health concern for countries with populations that are increasingly susceptible to this vector-borne disease, such as Sri Lanka. Almost 55,150 dengue cases were reported in Sri Lanka in 2016, with more than 30.40% of cases (n=16,767) originating from Colombo, which struggles with an outdated manual paper-based dengue outbreak management system. Community education and outreach about dengue are also executed using paper-based media channels such as pamphlets and brochures. Yet, Sri Lanka is one of the countries with the most affordable rates of mobile services in the world, with penetration rates higher than most developing countries.

**Objectives:**

To combat the issues of an exhausted dengue management system and to make use of new technology, in 2015, a mobile participatory system for dengue surveillance called Mo-Buzz was developed and launched in Colombo, Sri Lanka. This paper describes the system’s components and uptake, along with other similar disease surveillance systems.

**Methods:**

We developed Mo-Buzz and tested its feasibility for dengue. Two versions of the app were developed. The first was for use by public health inspectors (PHIs) to digitize form filling and recording of site visit information, and track dengue outbreaks on a real-time dengue hotspot map using the global positioning system technology. The system also provides updated dengue infographics and educational materials for the PHIs to educate the general public.

The second version of Mo-Buzz was created for use by the general public. This system uses dynamic mapping to help educate and inform the general public about potential outbreak regions and allow them to report dengue symptoms and post pictures of potential dengue mosquito–breeding sites, which are automatically sent to the health authorities. Targeted alerts can be sent to users depending on their geographical location.

**Results:**

We assessed the usage and the usability of the app and its impact on overall dengue transmission in Colombo. Initial uptake of Mo-Buzz for PHIs was low; however, after more training and incentivizing of usage, the uptake of the app in PHIs increased from less than 10% (n=3) to 76% (n=38). The general public user evaluation feedback was fruitful in providing improvements to the app, and at present, a number of solutions are being reviewed as viable options to boost user uptake.

**Conclusions:**

From our Mo-Buzz study, we have learned that initial acceptance of such systems can be slow but eventually positive. Mobile and social media interventions, such as Mo-Buzz, are poised to play a greater role in shaping risk perceptions and managing seasonal and sporadic outbreaks of infectious diseases in Asia and around the world.

## Introduction

Underreporting of *Aedes*-borne viruses is high. For instance, only 2 million cases of dengue fever are reported each year to the World Health Organization although an estimated 98 million symptomatic cases occur [1,2]. Engaging communities in the surveillance of dengue, Zika, and other *Aedes*-borne viral diseases has the potential to increase reporting and reduce the incidence and transmission of *Aedes aegypti* [3]. Dengue is a global disease, with around half the world’s population estimated to be at risk of infection. Incidence of dengue has increased over the past decades, with outbreaks rising in frequency, magnitude, and the number of affected countries [4,5]. It is estimated that 128 countries and 3.9 billion people are at risk of dengue [6,7]. The impact of dengue has been measured in terms of both monetary value, as well as public health metrics such as disability-adjusted life-years [8,9].

Dengue has been a national public health concern for Sri Lanka in recent years, with nearly 55,150 dengue cases reported in 2016. More than 30.40% (n=16,767) of these cases have originated from the western province of Colombo, which grapples with an exhausted dengue outbreak management system [10]. The problem is characterized by manual mechanisms of conducting surveillance (including identifying breeding sites), paper-based reporting of dengue cases to the hospitals, and challenges to coordinating health authorities, such as the Colombo Municipal Council (CMC), with public health inspectors (PHIs) and hospital epidemiological staff who find cases of dengue so that breeding sites can be treated [11]. Additionally, mapping of dengue hotspots during dengue outbreaks has been done reactively as each outbreak unravels, as opposed to relying on models to develop proactive mapping that can help both authorities and the public undertake preventive actions in advance.

Sri Lanka is one of the countries with the most affordable rates of mobile services in the world and has witnessed an unprecedented growth in the penetration of mobile services; its mobile phone penetration rates are higher than those in most developing countries [12]. However, dengue programs have yet to benefit from this technological trend, even as vast swathes of the Sri Lankan population become increasingly susceptible to this vector-borne disease. Community education and outreach about dengue continue to be executed using outdated media channels such as pamphlets and brochures. As a result, the capacity of public health institutions to persuade the public to practice healthy behaviors that protect from dengue is limited.

This paper describes the lessons and learning experiences from the development and launch of Mo-Buzz, a mobile participatory system for dengue surveillance, which was launched in Colombo, Sri Lanka, in 2015. Two versions of Mo-Buzz for dengue were launched: the first for use by PHIs and the second for the general public. Both versions are described here. For comparison and to highlight interest and growth in this type of surveillance system, we also describe other vector disease surveillance systems. A major objective of the development of Mo-Buzz and similar systems was to improve dengue surveillance and to reduce the spread of dengue, with the hope of expanding the technology to other infectious diseases.

## Methods

### Development of the System

Two forms of the Mo-Buzz mobile participatory system for dengue surveillance were developed in Colombo, Sri Lanka, which are described below. The research was approved by the institutional review board of the Nanyang Technological University for the protection of human subjects.

### Mo-Buzz for Public Health Inspectors

Before Mo-Buzz was developed for PHIs, the Mo-Buzz research team conducted a series of in-depth needs assessment interviews with Sri Lankan PHIs to ascertain informational and technological needs pertaining to dengue [13]. The interviews were recorded and transcribed by a professional, and a research assistant coded the data into NVIVO (QSR International), a statistical package used in qualitative analysis. The data were analyzed to derive themes relating to issues affecting the effectiveness of the work of the PHIs. The Mo-Buzz research team found several key challenges concerning the PHIs and then developed solutions for each key challenge.

First, PHIs reported that filling in lengthy forms was extremely tedious and time-consuming and often resulted in mistakes. The Mo-Buzz research team postulated that digitizing the process would decrease the time taken to fill in the forms and would reduce the number of errors made. Second, the PHIs were using a separate global positioning system (GPS) tag, which the researchers found to be woefully inaccurate. The Mo-Buzz research team proposed utilizing tablets that have in-built GPS technology with increased sensitivity. Third, the PHIs took photographs of dengue mosquito–breeding sites, which had to be transported manually, and they often reported that the photos were misplaced or damaged, making them inadmissible. The Mo-Buzz research team suggested using the in-built digital cameras in the smart devices to transmit the pictures onto a Web-based database automatically. Finally, the PHIs reported that many of the educational materials being used by them were outdated. The research team postulated that having updated infographics would serve as interesting and entertaining platforms for the PHIs to educate the general public on dengue [14].

**Figure 1:**
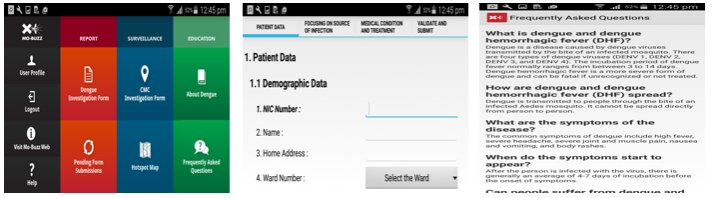
Screenshots of the Mo-Buzz for Public Health Inspectors application. Home page, Dengue Investigation Form (DIF), Frequently Asked Questions (FAQ) (left to right).

On the basis of the Mo-Buzz research team’s formative needs assessment interviews and the proposed solutions, the Mo-Buzz system that was developed for PHI use digitizes the three main roles of the PHIs: (1) recording field and site visit information (digitized surveillance), (2) keeping track of outbreak clusters in their regions (digitized dengue monitoring and mapping), and (3) providing dengue-related information to members of the general public (digitized dengue education). Each of these is described in turn below. Figure 1 shows the iPad (Apple Inc) view of the Mo-Buzz app for use by PHIs.

Digitized surveillance enables PHIs to record details of the case on a digital dengue investigation form (DIF). The digitized version of the form is clear and concise and has in-built alerts in the event that the PHI leaves a mandatory section blank. Both the DIFs and any photos of potential breeding sites taken by PHIs are automatically geotagged and can be shared with relevant health authorities with the press of a button.

Digitized dengue monitoring and mapping offers a real-time dengue hotspot map that is automatically updated when PHIs submit a DIF. The automatic geotagged breeding site reports (described above) are also represented on this same map. These map blips allow PHIs and relevant authorities to plan preventative interventions according to outbreak patterns, increasing both the efficacy and efficiency of their work.

Digitized dengue education seeks to increase positive interactions between the PHIs and the general public. The Mo-Buzz system offers PHIs information related to dengue in the form of entertaining infographics. Information is translated into the three main languages of Sri Lanka: English, Sinhalese, and Tamil. The educational materials include existing pamphlets handed out by the CMC and new information gathered from the Centers for Disease Control and Prevention.

### Mo-Buzz for the General Public

The general public version of Mo-Buzz was designed for the general local population. As with Mo-Buzz for PHIs, a formative research survey was conducted before deployment. A total of 513 members of the general public in Colombo were surveyed to assess the potential receptivity to the Mo-Buzz system for public use through baseline surveys [15].

Protection motivation theory, which suggests that an individual’s intention to perform behavior is intrinsically driven by the need to protect him or her from the health threat under consideration, was used as a framework in our research. Protection motivation theory regression models that are used to predict intention to use rely on variables such as perceived susceptibility and severity [16].

The survey found that the overall receptivity to the proposed Mo-Buzz system was high, with a score of greater than 4 on a 5-point scale. However, although the level of perceived severity of dengue was high with scores consistently greater than 4 when stratified by age, education, income, and ethnicity, the level of perceived susceptibility was low, with scores below 4 on a 5-point scale. In multivariate analyses, severity and susceptibility were found to be significant predictors of intention to use. We concluded that a social media–based system for dengue prevention would be positively received among Colombo residents, but that a targeted strategic health communication effort to raise dengue-related threat perceptions will be needed to encourage greater adoption and use of the system [17].

**Figure 2:**
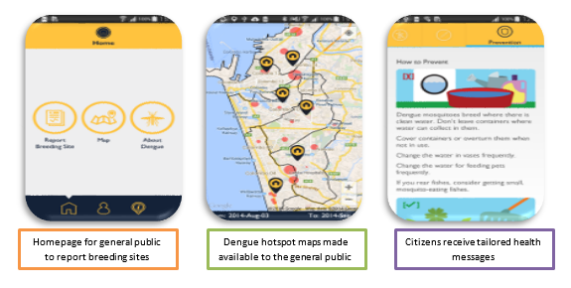
Screenshots of Mo-Buzz for the general public. Home page, hotspot mapping, health education materials (left to right).

The public version of Mo-Buzz integrated three components: (1) dynamic disease mapping, (2) civic engagement, and (3) health communication. Each of these is briefly described below. More detailed descriptions are available elsewhere [18]. Screenshots of the Mo-Buzz public version app are shown in Figure 2.

Dynamic mapping helps to inform the general public about potential outbreak regions using an algorithm and computer simulation that utilizes both archival and current dengue-related data. The purpose of the component is to forewarn stakeholders (eg, health authorities) and members of public in the form of hotspot mapping, and it was designed to facilitate the planning of preventative measures and more efficient and effective management of resources.

The civic engagement component was developed because in the past, disease surveillance during an epidemic or pandemic has been the sole preserve of epidemiological divisions. The Mo-Buzz research team endeavored to develop a two-way platform built on the concept of crowdsourcing that allows public health officials to not only transmit information but also receive real-time intelligence on the spread of disease. Specifically, Mo-Buzz allows the general public to report dengue symptoms and/or post pictures of potential dengue mosquito–breeding sites. As with Mo-Buzz for PHIs, such reports and postings are automatically geotagged and sent to the health authorities with a click of a button, thereby stimulating the first stage of response by health authorities.

The health communication component consists of two modules. The first module is static and contains health education materials that discuss dengue transmission, symptoms, treatment, and prevention. The second module is dynamic and includes targeted alerts that are sent to users, depending on a user’s geographical status and predictions developed by the hotspot maps.

## Results

### Mo-Buzz for Public Health Inspectors

When the Mo-Buzz for PHIs system was about 8 months into deployment by the CMC, the Mo-Buzz research team conducted follow-up in-depth interviews with 16 PHIs to ascertain the efficacy of Mo-Buzz.

The researchers found that Mo-Buzz reduced the amount of time taken to fill out and submit the dengue forms. Previously, this was a lengthy process that took 7 to 10 days and involved various stages of sending paperwork to all the relevant agencies; however, Mo-Buzz enabled PHIs to send information in real time at the click of a button, expediting the whole process to 2 to 3 days. However, minor tweaks still needed to be made, such as providing a place on the form to report specific ages of clients in years and months. GPS technology was found to be far more accurate than the previous mode of geotagging, and minor glitches occurred far more infrequently. The PHIs noted that the dynamic disease mapping component (ie, hotspot mapping), although useful for them, was not deemed necessary for the public. They were worried that it might trigger outbreaks of panic. However, the general public was receptive to this component. The PHIs also mentioned that the educational materials were useful but could be more interactive and that the in-built camera needed to be of better quality and include flash, as most breeding sites were in areas with poor lighting.

Communication with stakeholders (specifically, CMC, which employs PHIs, and Mobitel and the University of Colombo, which recruit general public users) was problematic, as the Mo-Buzz research team was based in Singapore. The Mo-Buzz research team overcame this issue by learning about Sri Lankan customs and culture from Sri Lankans living in Singapore and by undertaking field trips and interviews. These activities allowed researchers to understand the people of Sri Lanka better. Stakeholder communication was made more difficult by differences in opinions borne of views that were influenced by different specialties. For example, although the Mo-Buzz research team offered expertise in health communication, psychology, behavioral science, and computer science, collaborators from the CMC were medical physicians. However, the openness of both parties to new and different ideas vastly facilitated discussions on the implementation of Mo-Buzz.

The Mo-Buzz research team ran into several difficulties during baseline interviews with the PHIs. First, language posed a barrier. The main mode of communication in Sri Lanka is Sinhalese or Tamil, but none of the researchers were proficient in either language. As such, an external translator who was fluent in Sinhalese, Tamil, and English was employed to overcome this language barrier.

Regarding uptake of Mo-Buzz, the Mo-Buzz research team found that PHIs were initially resistant and that most were not mobile technology literate. Thus, initial uptake of Mo-Buzz was low, as roughly less than 10% (n=3) of PHIs were utilizing the technology. This was a cause for concern because Mo-Buzz was developed to simplify the duties of the PHIs and streamline the epidemiological process. Even some PHIs who were mobile literate were not inclined to take up a technological approach to submitting reports, as there was an innate fear and distrust of technology. As a solution, CMC decided to send one of the more technically proficient PHIs to Singapore to work with the researchers and learn the system well. The PHI who was sent was also tasked with troubleshooting issues faced by other PHIs. On learning the system, the PHI returned to Colombo as an ambassador to train his fellow PHIs and help promote the usage and troubleshoot. Following this training, uptake of the app increased.

**Figure 3:**
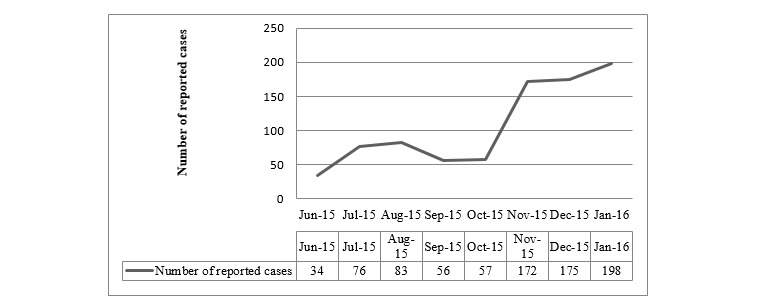
Public Health Inspector usage of mobile Mo-Buzz app for reporting dengue cases over time.

Another method implemented by the CMC to increase uptake of Mo-Buzz was to incentivize reports submitted by the PHIs (ie, from October 2015 onward). The total number of reports submitted by a PHI was taken into account when considering the PHI’s yearly performance bonuses and pay increments. These methods caused a massive rise in users, with 38 of the 50 PHIs (76%) using Mo-Buzz to submit reports (see Figure 3). The remaining 12 PHIs were unable to use the app because either the tablets issued were unusable or the PHIs were not issued a tablet.

Conducting postintervention evaluation proved tricky but important, as a representative from the Mo-Buzz research team had to make a separate trip to Colombo to conduct the interviews together with the translator. The researcher was able to conduct only 16 interviews in total, across a period of 4 days. A faculty member of the University of Colombo School of Computing assisted in translating and transcribing the interview sessions. Feedback from the PHIs proved to be invaluable and led toward significant redevelopment and upgrading of the existing Mo-Buzz for PHI application.

In 2015, it was reported that the dengue cases in Colombo have reduced to less than 9881 [19]. These findings, as well as positive feedback from the CMC, indicate that the Mo-Buzz system has helped to bring down about one-third of dengue cases compared with the number of cases in 2014.

### Mo-Buzz for the General Public

The Mo-Buzz research team had taken great stock in feedback given by members of the public who were surveyed during the conducting of the baseline surveys. However, there were several challenges faced by the Mo-Buzz research team during the various phases of the development of Mo-Buzz for the general public.

Baseline surveys proved difficult to conduct, as most of the general public was unwilling to participate in the survey. The researchers had to conduct baseline surveys on the public via word of mouth using the aid of students, staff, and faculty members of the University of Colombo, People’s Bank, Mobitel, and Rupavahini Corporation. Finally, the researchers were able to recruit 513 members of the general public to participate, which helped to provide vital information in the development of the public system.

After integrating knowledge provided from the baseline survey, a pilot test of the public version of Mo-Buzz system was launched among 80 working adults and local university students. After 1 week of use, they were given a feedback survey. Participants were found to be receptive to the app, with 93.8% (n=481) saying they would use the app when it was launched. On the basis of the feedback, the Mo-Buzz app included up-to-date dengue education materials for the perusal of the general public. Efforts were also made to make the app more interactive for the users, and graphic designers were utilized to create interesting infographics for users to peruse. The pilot study participants also provided ideas on how to boost participation, such as providing 1500 Sri Lankan rupees (about US $10) as tokens of appreciation.

Using the baseline survey and pilot test data to improve the system, initial efforts were made to avail the Mo-Buzz app for mass usage in Colombo; however, launching of the system was delayed as a political election was underway during the agreed-upon launch date, which could have had implications for government approvals.

A soft launch of the app was then undertaken, where users within the general population were allowed to download the public version of Mo-Buzz. The number of users was low with approximately 50 participants downloading and using the app. So, the Mo-Buzz research team partnered with communication students from our university in Singapore to conduct additional research to develop marketing and advertising solutions to spread awareness of Mo-Buzz. Then a fieldwork assessment was conducted in late 2015. At present, the team is working with CMC and Mobitel by hosting a hackathon event with the hopes of connecting technologists, designers, and public health experts to reassess and ideate the public launch strategy and app content.

## Discussion

### Principal Findings

Mobile and social media interventions, such as Mo-Buzz, are poised to play a greater role in shaping risk perceptions and managing seasonal and sporadic outbreaks of infectious diseases in Asia and around the world. An example of a comparable participatory surveillance system for dengue and other mosquito-borne infectious diseases is Kidenga, a US-based system, described below.

Mo-Buzz is one of several community-based surveillance systems designed to engage the public in reporting mosquito activity and syndromes consistent with *Aedes*-borne diseases. Other systems include Kidenga, which targets the United States-Mexico border region, SaludBoricua in Puerto Rico, and Dengue Na Web in Brazil. These systems differ in their approach to engagement and data collection, but all share a common goal of detecting and informing the public about dengue transmission.

Kidenga is a mobile community-based syndromic surveillance app available exclusively on Android and iPhone mobile phones released in 2016. It was developed to detect individuals with symptoms suggestive of dengue, Zika, and Chikungunya virus infections and to track the activity of their vectors, *Ae aegypti* and *Ae albopictus*. Currently, Kidenga targets populations in three high-risk states: Arizona, Florida, and Texas [20]. The objectives of the app, similar to Mo-Buzz, were threefold: (1) to detect potential transmission, (2) to educate the public about existing disease trends and disease and mosquito prevention and control, and (3) to provide education to clinicians. Each week, a push notification is sent to users to notify them to report symptoms of illness, travel history, and mosquito activity. When an individual reports that she, he, or a family member has been ill, this triggers two educational messages to the user’s email address on file, which include: (1) prevention and control strategies directed to the user and (2) treatment and testing information directed to clinicians the user may see. Within the app, such as with Mo-Buzz, maps of user-generated data, confirmed case counts from public health partners, mosquito distribution maps, and information about prevention and control strategies are accessible for users to browse and to learn more. Additionally, current news on the diseases and vectors are provided on an active news feed. These components add value to the app for users. However, identifying standard sources of data for confirmed cases can prove challenging in countries with decentralized health systems, such as the United States. Data must be accessed for each state separately, as formats and accessibility differ widely. Additional functionality of Kidenga may include a similar interface to Mo-Buzz for community health workers, a linked mosquito-hunt game to sustain interest and targeted weather-based mosquito warnings to personalize messaging and to motivate action.

SaludBoricua and Dengue Na Web are both Web-based systems that are marketed through social marketing and traditional channels to enroll users to report symptoms on a weekly basis [21,22]. Dengue Na Web is distinguished as the first community-based syndromic system designed to track a non-influenza-like syndrome. This system is used in Salvador de Bahia, Brazil. Established in 2011, it has received over 2 million visitors at the time of writing this paper. Individuals are depicted as suspected dengue, other symptoms, no symptoms, or no report. General information on dengue, its symptoms, and its prevention are further included as Web content. Mosquito activity is not tracked by Dengue Na Web, and confirmed case information is not presented. Similarly, SaludBoricua uses a Web-based interface. SaludBoricua was developed as a partner system to the FluNearYou system for detecting influenza activity. In addition to influenza, SaludBoricua is being used to monitor symptoms reported by users for dengue and leptospirosis. Minimal information is presented within the Webpage, but links to public health information from organizations such as the Pan American Health Organization provide general information to users about the diseases under surveillance. One particularly valuable feature of SaludBoricua is news items tailored to the region and diseases prevalent in that region.

These complementary systems are all at different stages of development and have relative strengths and weaknesses. Lack of a community–health worker interface may minimize the reach of these other systems, which could be considered in next phases. In addition, a mobile phone–based platform, such as Kidenga, may bias users toward higher income groups and not fully represent the target population. All of these systems will require a rigorous evaluation process to determine whether they fulfill the criteria of a useful surveillance system, including positive predictive value, sensitivity, acceptability, flexibility, data quality, simplicity, representativeness, and timeliness [23].

### Conclusion and Future Development

After general population baseline surveys and discussions with PHIs, the Mo-Buzz app was developed for and tested among two groups: PHIs and the general public. Overall, the PHIs were receptive of the system, and after facing several challenges, the uptake and reporting of dengue increased. However, although the general public was originally receptive of the idea of Mo-Buzz, the uptake and engagement with the app remain low; solutions for this are currently being discussed.

Mo-Buzz, Dengue Na Web, SaludBoricua, and Kidenga represent novel interventions for mosquito-borne diseases where social media is being used not only for disseminating persuasive health messages but also for bolstering the health services infrastructure and creating real-time or near real-time links between the general public and health authorities. These apps, as previously mentioned, follow the same overall targets: detection of potential transmission, educating the public about the disease and vector control measures, and providing education to clinicians. It is anticipated that these types of systems will increase when a history of effectiveness is established. Several potential future directions for these systems include the expansion of the app’s usage to other diseases. Currently, SaludBoricua and Kidenga are being used to monitor additional syndromes indicative of influenza and leptospirosis (SaludBoricua) and Zika and Chikungunya (Kidenga). Although the value of these systems may increase with expansion to viruses in addition to dengue, this can lead to additional challenges. For example, using these systems to monitor Zika-like illness may be especially problematic given the challenges because of vague presentation of symptoms and in developing a syndrome definition with sufficient specificity to warrant public health action. Inclusion of other diseases also makes the creation of educational materials more challenging.

From our Mo-Buzz study, we have learned that initial acceptance of such systems can be slow but eventually positive. We predict that future adoption and sustained use will be driven by a complex set of variables at different levels of the social ecosystem, such as social trust and willingness to share information. On the basis of previous research, we foresee that passive (viewing hotspot maps) and active (reporting dengue hotspot) participation might be driven by different kinds of variables [24]. To boost participation in Mo-Buzz, we are now in the process of launching a communication campaign to create greater awareness and uptake of the app. Future work will focus on expanding the capabilities of Mo-Buzz to address other health problems in Sri Lanka and around the region and integrating its capabilities with the larger eHealth information architecture in the region, as well as building upon existing technologies to create surveillance systems catering to other diseases [25].

## Appendix

Multimedia Appendix 1 Dengue Investigation Form (DIF) utilized by Public Health Inspectors (PHIs) in Colombo, Sri Lanka.

## Abbreviations

CMC: Colombo Municipal Council
DIF: dengue investigation form
GPS: global positioning system
PHIs: public health inspectors
