# Supplementary material for: Lessons From the Implementation of Mo-Buzz, a Mobile Pandemic Surveillance System for Dengue
Source: JMIR Public Health Surveill. 2017 Oct 2;3(4):e65. doi: 10.2196/publichealth.7376 (PMC5643840; doi:10.2196/publichealth.7376)

Appendix A

Dengue Investigation Form (DIF)

1. Patient Data

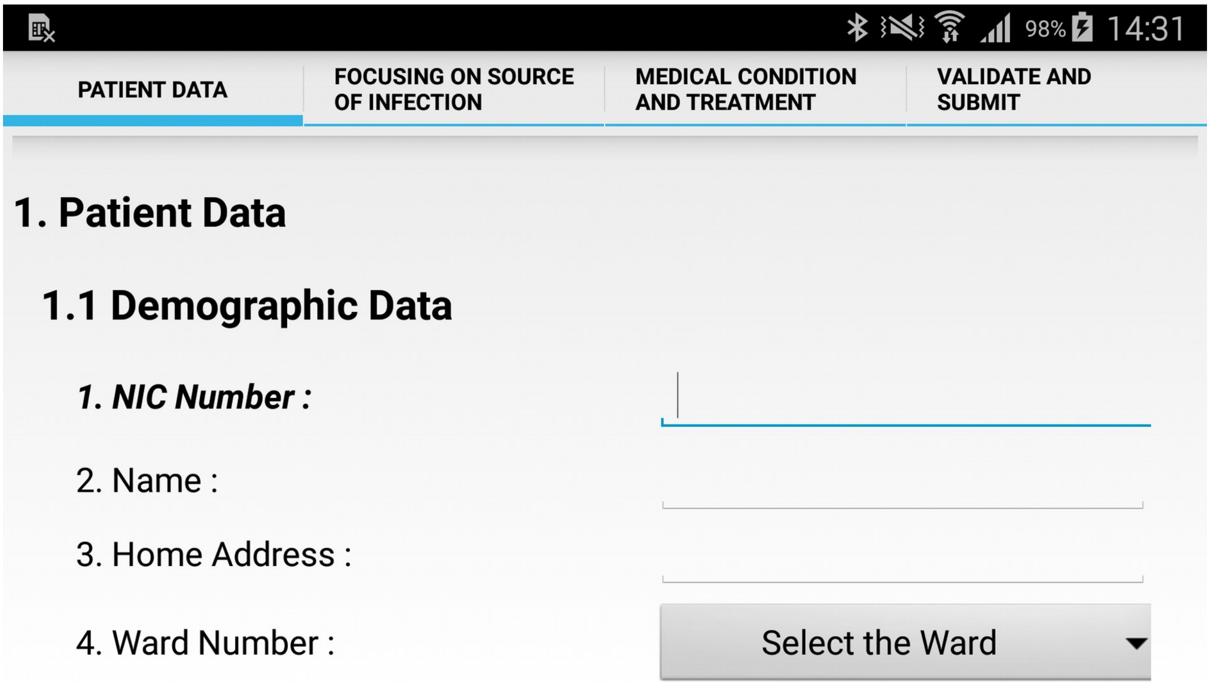

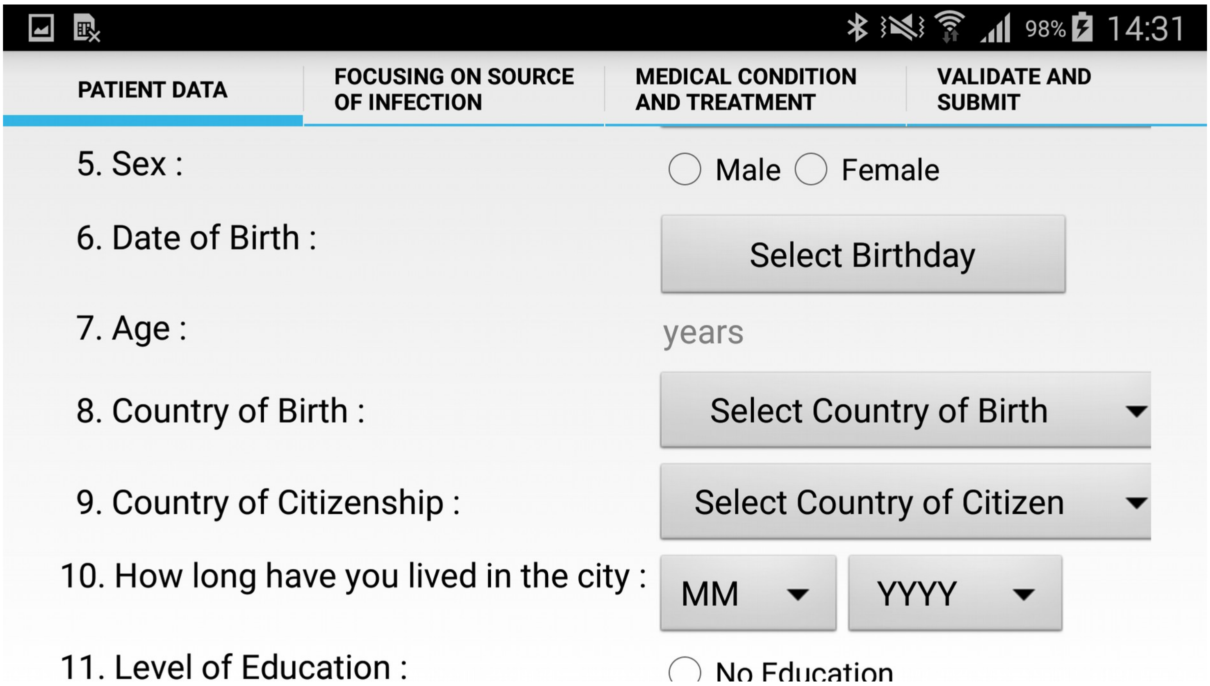

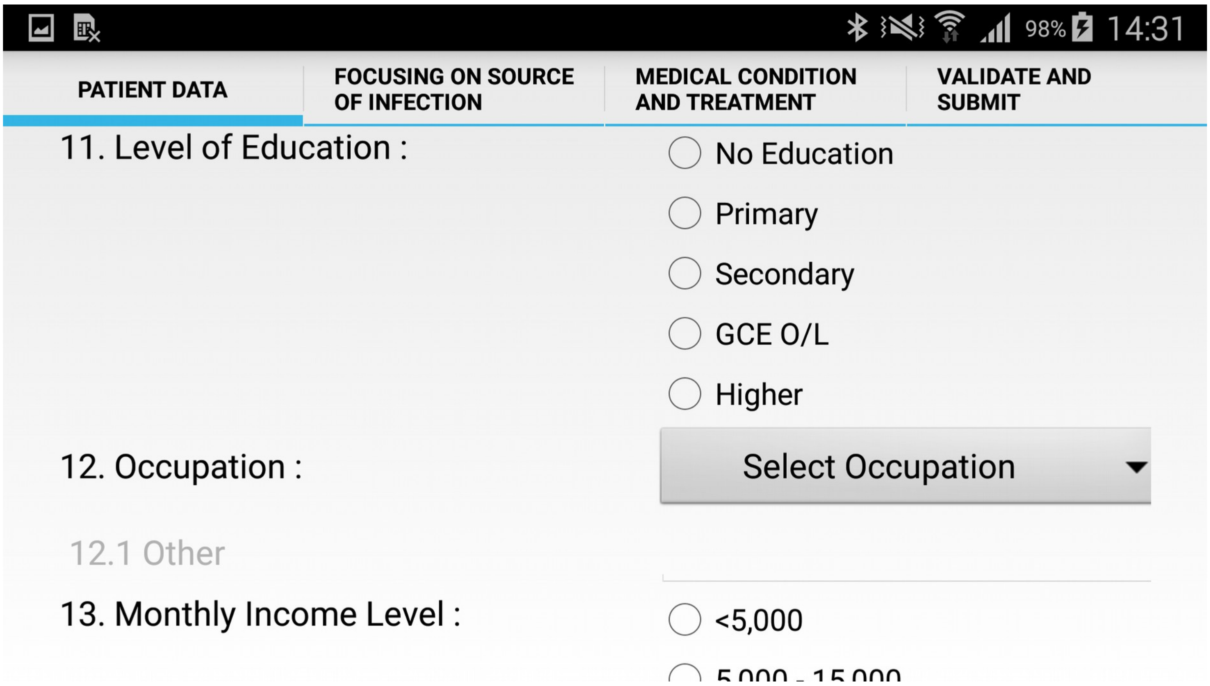
98%
14:31
PATIENT DATA
FOCUSING ON SOURCE OF INFECTION
MEDICAL CONDITION AND TREATMENT
VALIDATE AND SUBMIT
11. Level of Education :
No Education
Primary
Secondary
GCE O/L
Higher
12. Occupation :
Select Occupation
12.1 Other
13. Monthly Income Level :
<5,000

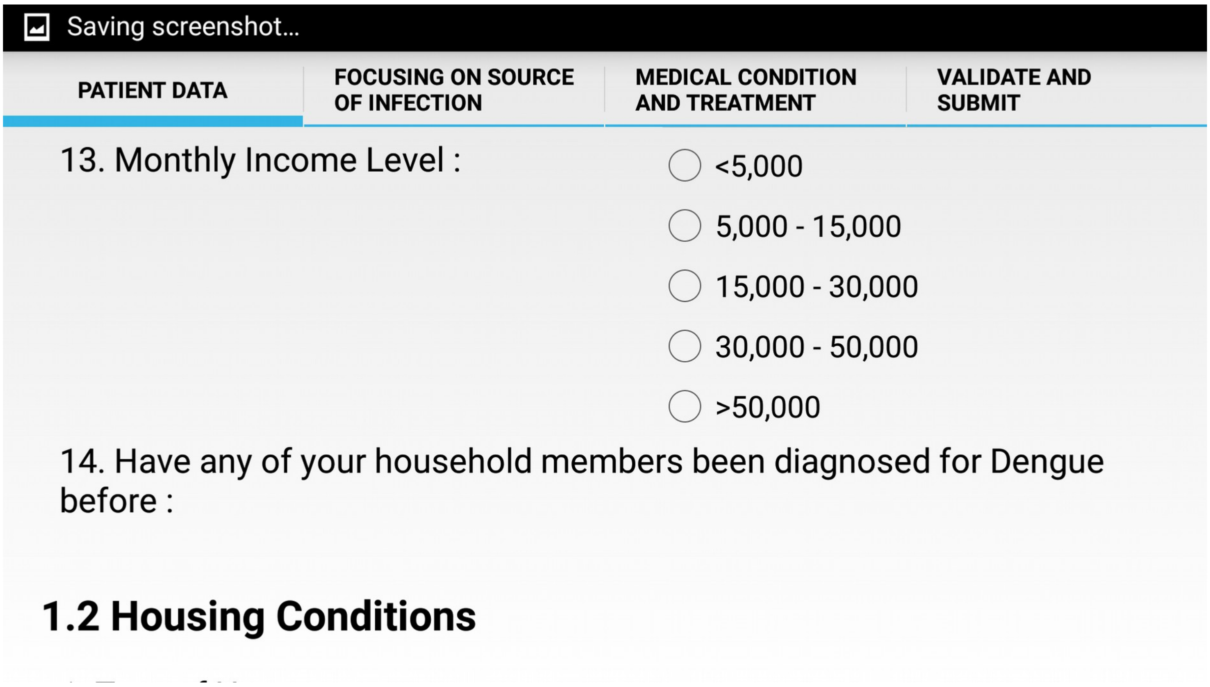
Saving screenshot...
PATIENT DATA
FOCUSING ON SOURCE OF INFECTION
MEDICAL CONDITION AND TREATMENT
VALIDATE AND SUBMIT
13. Monthly Income Level :
<5,000
5,000 - 15,000
15,000 - 30,000
30,000 - 50,000
>50,000
14. Have any of your household members been diagnosed for Dengue before :
1.2 Housing Conditions

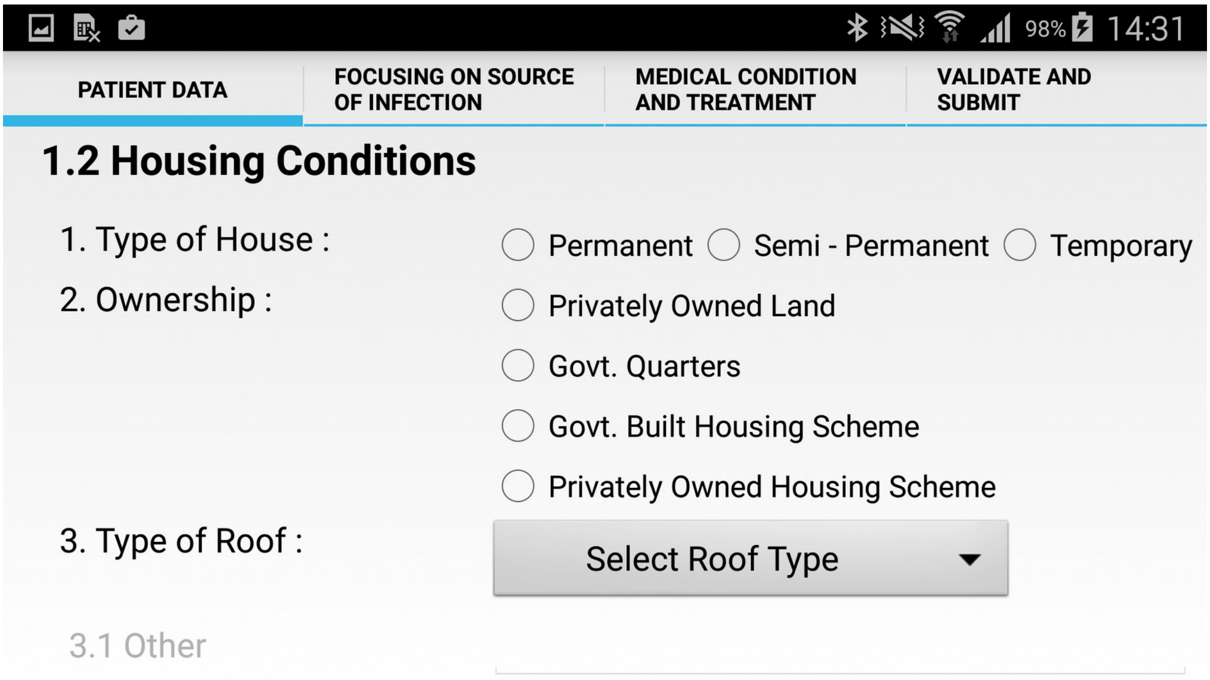
98% 14:31
PATIENT DATA
FOCUSING ON SOURCE OF INFECTION
MEDICAL CONDITION AND TREATMENT
VALIDATE AND SUBMIT
1.2 Housing Conditions
1. Type of House :
Permanent
Semi - Permanent
Temporary
2. Ownership :
Privately Owned Land
Govt. Quarters
Govt. Built Housing Scheme
Privately Owned Housing Scheme
3. Type of Roof :
Select Roof Type
3.1 Other

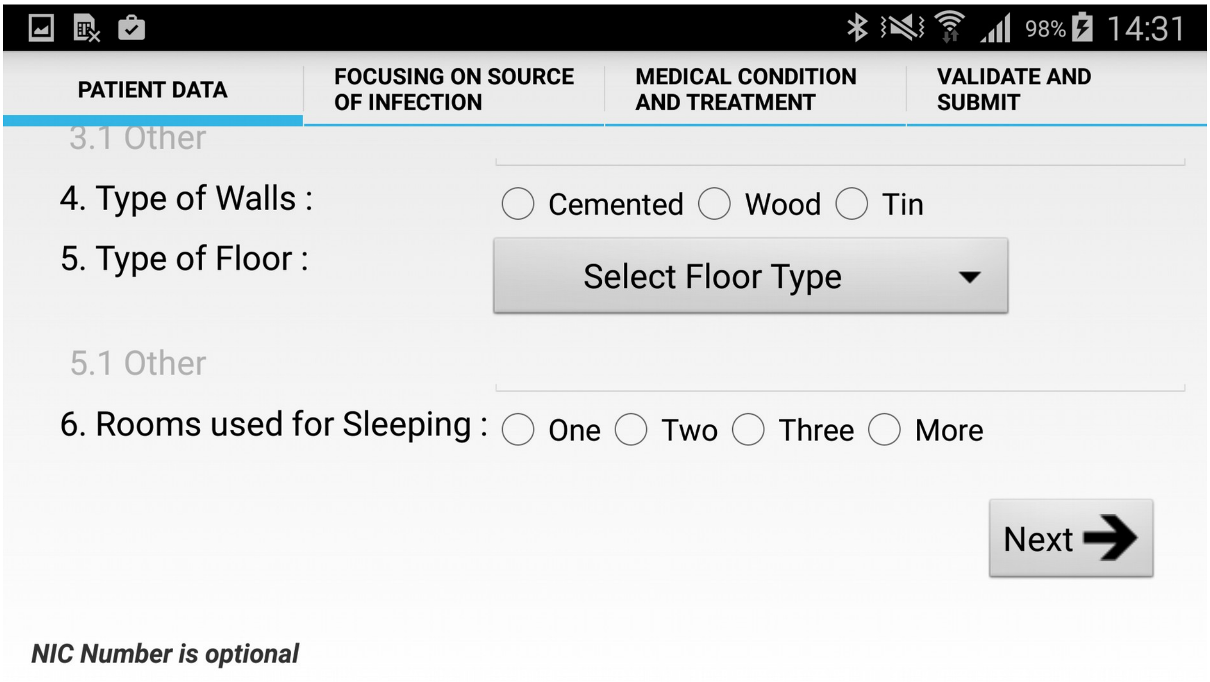
98% 14:31
PATIENT DATA
FOCUSING ON SOURCE OF INFECTION
MEDICAL CONDITION AND TREATMENT
VALIDATE AND SUBMIT
3.1 Other
4. Type of Walls :
Cemented
Wood
Tin
5. Type of Floor :
Select Floor Type
5.1 Other
6. Rooms used for Sleeping :
One
Two
Three
More
Next
NIC Number is optional

2. Source of Infection

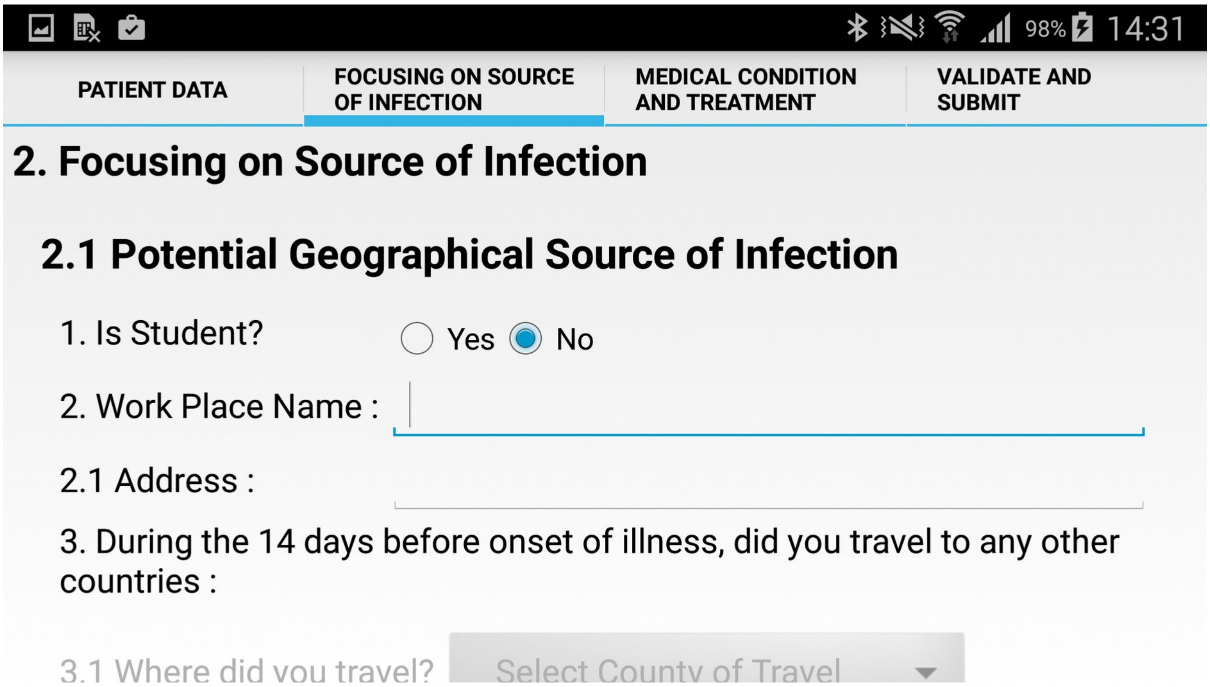

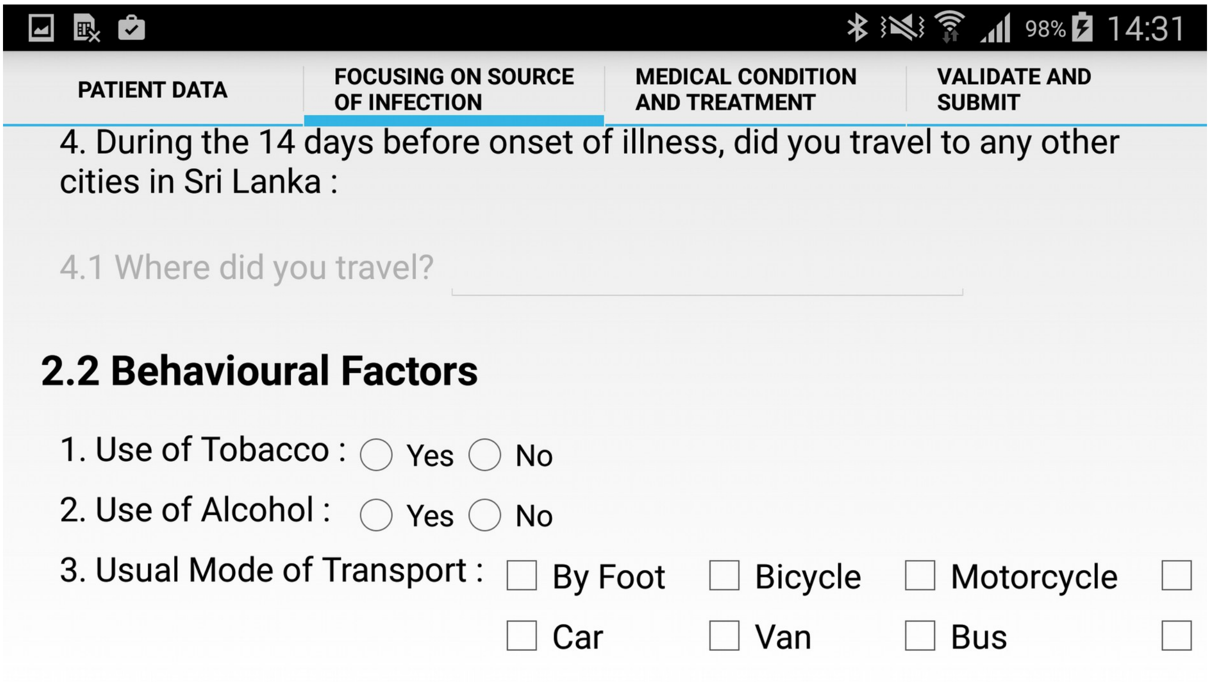

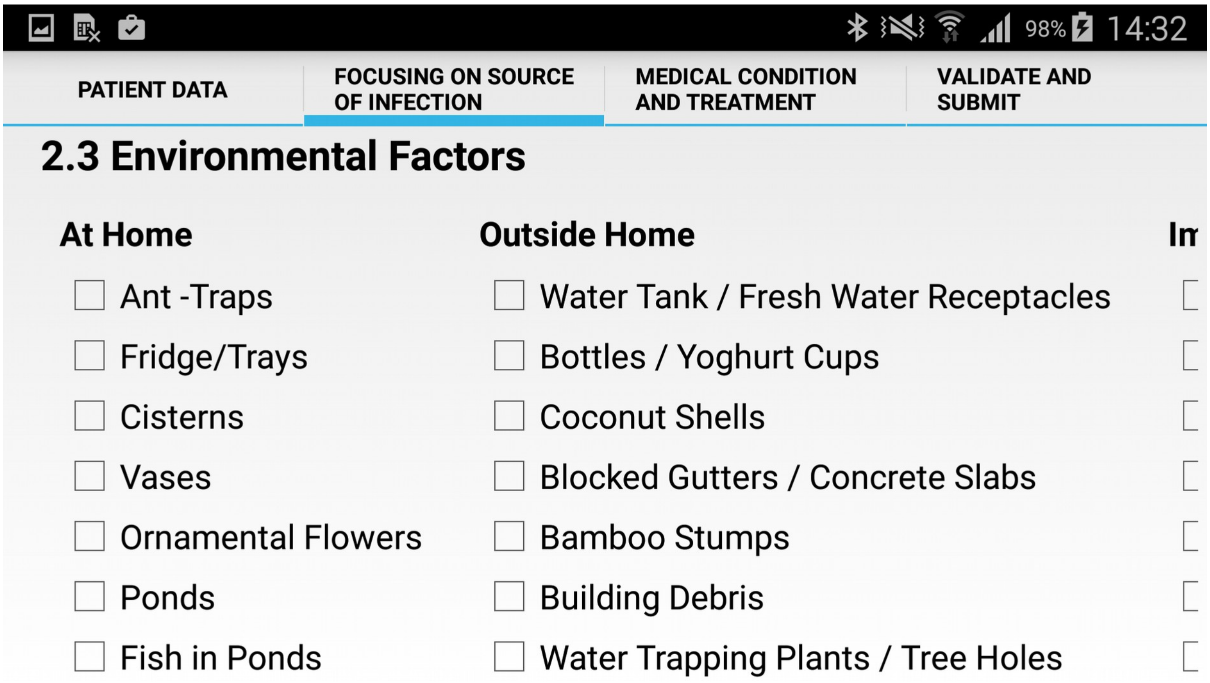

3. Medical Condition and Treatment

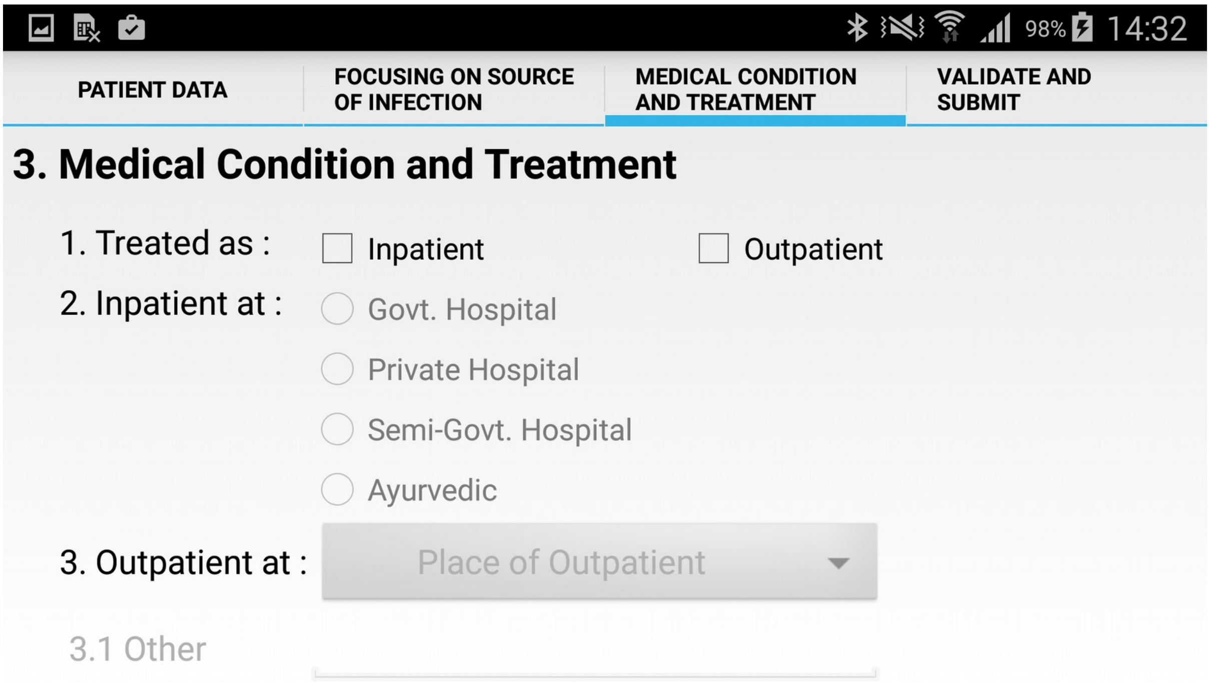

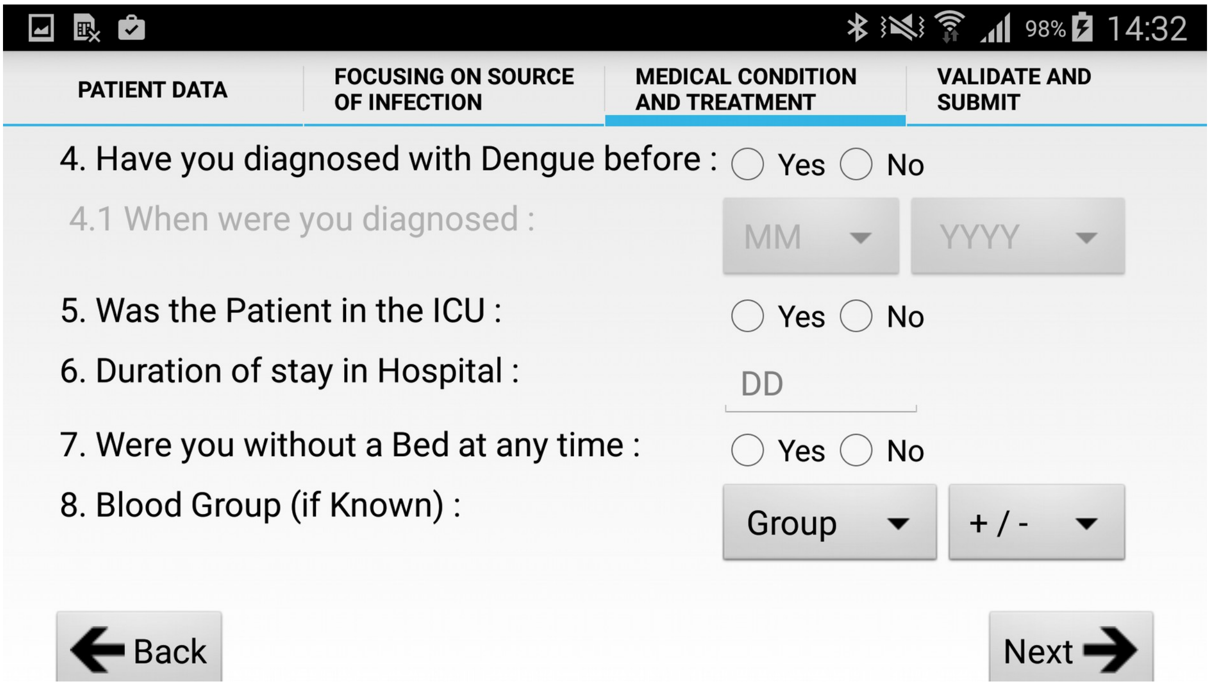

4. Validation and Submission

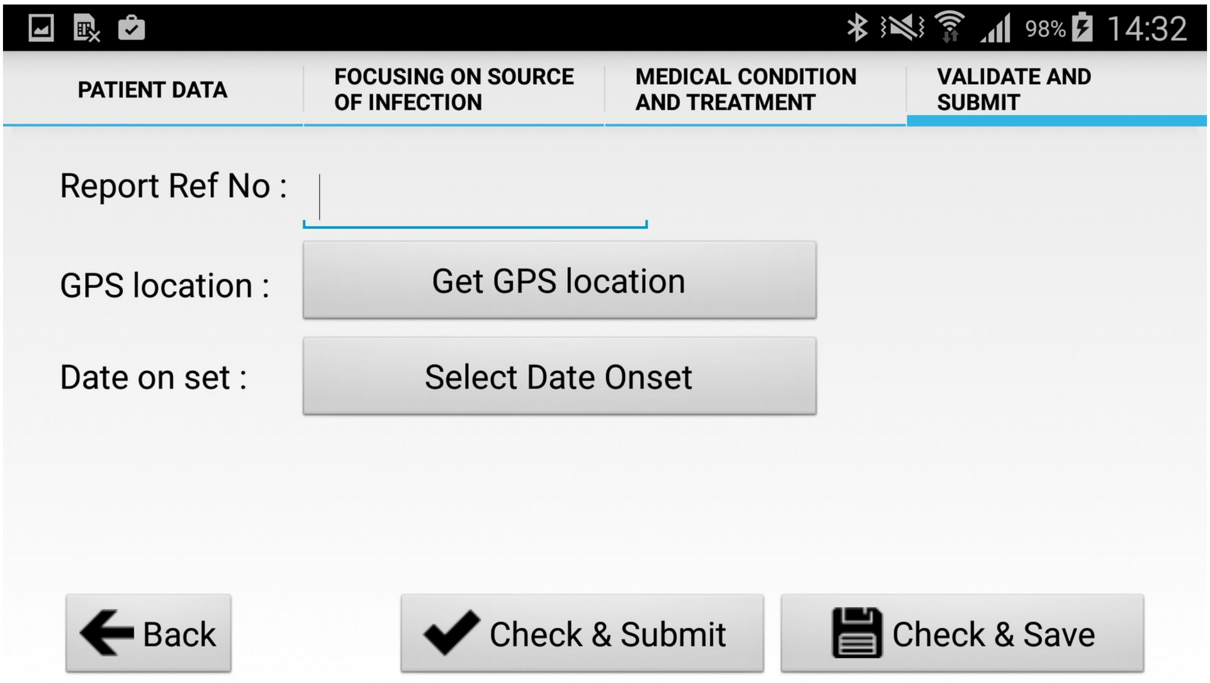

Supplement: Multimedia Appendix 1 [file publichealth_v3i4e65_app1.pdf]
